# Risk Factors and Postoperative Complications of Lobectomy for Non-Small Cell Lung Cancer: An Exploratory Analysis of Premedication and Clinical Variables

**DOI:** 10.3390/medicina60122088

**Published:** 2024-12-20

**Authors:** Markéta Kepičová, Lubomír Tulinský, Adéla Kondé, Paula Dzurňáková, Peter Ihnát, Dávid Adamica, Čestmír Neoral, Lubomír Martínek

**Affiliations:** 1 Department of Surgery, University Hospital Ostrava, 17. listopadu 1790, 708 52 Ostrava, Czech Republic; marketa.kepicova@fno.cz (M.K.); peter.ihnat@fno.cz (P.I.); david.adamica@fno.cz (D.A.); lubomir.martinek@fno.cz (L.M.); 2 Department of Surgical Studies, Faculty of Medicine, University of Ostrava, Syllabova 19, 703 00 Ostrava, Czech Republic; cestmir.neoral@osu.cz; 3 Department of Applied Mathematics, Faculty of Electrical Engineering and Computer Science, VSB—Technical University of Ostrava, 17. listopadu 2172/15, 708 00 Ostrava, Czech Republic; adela.konde@vsb.cz; 4 Department of Deputy Director for Science, Research and Education, University Hospital Ostrava, 17. listopadu 1790, 708 52 Ostrava, Czech Republic; 5 Department of Anesthesiology and Intensive Care Medicine, University Hospital Ostrava, 17. listopadu 1790, 708 52 Ostrava, Czech Republic; paula.dzurnakova@fno.cz

**Keywords:** lung cancer, lobectomy, risk factors, premedication, postoperative pneumonia, postoperative complication, thoracic surgery

## Abstract

*Background and Objectives*: Postoperative pneumonia and complications significantly impact outcomes in thoracic surgery, particularly for patients undergoing lobectomy for non-small cell lung cancer (NSCLC). This study evaluates whether preoperative premedication influences the risk of postoperative pneumonia and overall complications. *Materials and Methods*: This retrospective study included 346 patients who underwent lobectomy for NSCLC at the University Hospital Ostrava between 2015 and 2021. Data on demographic variables, tumour staging, surgical approach, and premedication (anticholinergics, benzodiazepines, antihistamines, and analgesics) were analysed. Postoperative outcomes included pneumonia and complications classified by the modified Clavien–Dindo system. *Results*: Premedication was not significantly associated with postoperative pneumonia (10.7%) or overall complications (26.0%). Tumour size was the only factor significantly associated with complications, with larger tumours increasing the odds (OR: 1.16, *p* = 0.032). Other factors, including age, ASA classification, BMI, and surgical approach, did not demonstrate significant associations with postoperative outcomes. *Conclusions*: Premedication does not appear to significantly influence the risk of postoperative pneumonia or overall complications in patients undergoing lobectomy for NSCLC. Similarly, other clinical variables, such as age, ASA classification, BMI, and surgical approach, also did not show significant associations with these outcomes. These findings suggest that premedication can be individualised without increasing postoperative risks. However, tumour size emerged as a significant factor associated with complications, highlighting the need for careful preoperative assessment and planning, particularly in patients with larger tumours.

## 1. Introduction

Lung cancer is one of the most common malignancies globally, with non-small cell lung cancer (NSCLC) accounting for the majority of cases [1,2,3,4]. Recent epidemiological data from the Czech Republic indicate an incidence of approximately 70 cases per 100,000 population, with a male predominance [2]. Surgical resection remains the cornerstone of curative treatment, encompassing techniques such as segmentectomy, lobectomy, and pneumonectomy. Minimally invasive approaches, including video-assisted thoracoscopic surgery (VATS) and robotic-assisted thoracoscopic surgery (RATS), are increasingly adopted due to their favourable postoperative outcomes [5,6,7,8,9,10].

Despite advances in surgical techniques and perioperative care, patients undergoing lung resection for NSCLC remain vulnerable to postoperative complications, particularly pneumonia, which is associated with significant morbidity and mortality. Numerous factors contribute to postoperative risks and are generally divided into three categories: (1) patient-related factors, (2) surgical technique-related factors, and (3) perioperative management factors [5,11,12].

Emerging evidence suggests that premedication, an integral part of perioperative care, could have a nuanced impact on postoperative outcomes [12,13]. While its benefits in reducing anxiety and facilitating anaesthetic induction are well documented, studies in other surgical disciplines, such as abdominal and orthopaedic surgery, have hinted at potential associations between preoperative pharmacological regimens and postoperative complications [13,14]. However, in thoracic surgery, such data remain scarce, and the interplay between premedication and complications like pneumonia warrants further investigation.

Premedication plays a critical role in the preparation of patients undergoing surgery. Administered during the pre-anaesthesia phase, it aims to reduce anxiety, alleviate pain, minimise salivation, and ensure a smoother induction of anaesthesia [12]. While these medications are essential for optimising surgical conditions, they are also associated with significant side effects, such as respiratory depression, excessive sedation, and altered haemodynamics, which may impact perioperative outcomes [12,13].

Recent studies have started to explore the relationship between premedication and postoperative complications in thoracic surgery, particularly in patients undergoing lung resection for NSCLC [12,13]. However, the evidence remains sparse and inconclusive [15,16]. Most investigations focus on immediate anaesthetic effects rather than long-term postoperative risks, such as pneumonia. This gap highlights the need for further research to evaluate how premedication regimens contribute to postoperative outcomes, especially in high-risk patients or those undergoing minimally invasive procedures [13,14]. A better understanding of these dynamics can help inform clinical decision-making and enhance perioperative management strategies.

This study aims to address this gap by evaluating the impact of preoperative clinical management—specifically premedication—on the risk of postoperative complications, with a primary focus on pneumonia, in patients undergoing lobectomy for NSCLC.

## 2. Materials and Methods

### 2.1. Design and Setting

This comparative study evaluated the effect of premedication on the risk of postoperative complications in patients undergoing lung resection for non-small cell lung cancer (NSCLC). Conducted at the University Hospital Ostrava, Czech Republic, it included all adult patients who underwent lobectomy between 1 January 2015 and 31 December 2021. Patients were excluded if they had lung metastases, carcinomatosis, benign tumours, or incomplete data. Lobectomies were performed using either thoracotomy or minimally invasive techniques (multiportal or uniportal VATS).

Data were collected and processed anonymously using the hospital information system. This study was approved by the ethics committee of the University Hospital (approval number FNOs/2024) and conducted following the Declaration of Helsinki. All participants provided informed consent, which included consent to the use of their medical records in future research and publication.

As our study was intended to provide an exploratory analysis, we focused on univariate analyses to assess the relationship between premedication and postoperative pneumonia. The univariate findings provide an initial understanding of the potential risk factors in this patient population.

### 2.2. Perioperative Care and Surgical Technique

Preoperative preparation included the administration of premedication approximately 30–60 min before surgery. The choice of premedication was guided by anaesthesiologists based on individual patient profiles and included benzodiazepines (e.g., midazolam or tofisopam), anticholinergics (e.g., atropine), antihistamines (e.g., promethazine), or analgesics (e.g., piritramide or morphine). Dosages were standardised within typical ranges: benzodiazepines at 3–7.5 mg, anticholinergics at 0.5–1 mg, antihistamines at 25–50 mg, and analgesics at 3–15 mg. Specific doses were individualised based on patient characteristics, weight, age, and pre-existing conditions, with the final dosage determined by the attending anaesthesiologist to balance effective sedation and anxiolysis with minimal side effects. All patients received antibiotic prophylaxis with cephazolin, or clindamycin/ciprofloxacin in cases of allergy, before surgical incision. Selective endotracheal intubation was employed to ensure one-lung ventilation.

The intraoperative approach was consistent across the cohort, utilising minimally invasive techniques (uniportal or multiportal VATS) or thoracotomy as appropriate. Postoperative care adhered to standardised protocols, including respiratory rehabilitation and chest drain management, to optimise recovery.

### 2.3. Data Collection

The dataset comprised demographic and clinical variables obtained from the hospital’s information system. Premedication included four classes of drugs: anticholinergics (e.g., atropine), benzodiazepines (e.g., midazolam, tofisopam), antihistamines (e.g., promethazine), and analgesics (e.g., piritramide, morphine). Patients who did not receive premedication were excluded from the analysis.

Demographic data included age, gender, body mass index (BMI), American Society of Anaesthesiologists (ASA) classification, tumour characteristics (size, histology, pTN stage), and surgical approach (thoracotomy, multiportal, or uniportal VATS). Clinical outcomes included operative time, hospital stay duration, and complications classified using the modified Clavien–Dindo system [17]. Special emphasis was placed on pneumonia, evaluated according to European Perioperative Clinical Outcome (EPCO) definitions [18].

### 2.4. Statistical Analysis

Numerical data are reported as medians and interquartile ranges (IQR), and categorical variables as absolute and relative frequencies (%). The assumptions of normality were tested using the Shapiro–Wilk test, and non-parametric methods were chosen where appropriate. The Kruskal–Wallis test (with Dunn’s post hoc analysis) and the Chi-square test of independence for contingency tables were used for comparisons. Univariate logistic regression models assessed the relationship between risk factors and outcomes, providing odds ratios, confidence intervals, and forest plots. Analyses were conducted using R software (version 4.3.1), with significance set to 0.05.

## 3. Results

A total of 346 patients were included in the analysis, comprising 212 men (61.3%) and 134 women (38.7%). The median age was 69 years (IQR: 63–73), and the median BMI was 27.8 kg/m^2^ (IQR: 24.8–31.1). Most patients were classified as ASA I or II (50.3%, n = 174), followed by ASA III (48.8%, n = 169), with a small proportion in ASA IV and V (0.9%, n = 3). One patient was excluded due to incomplete data.

Lobectomies were performed using the VATS technique in 64.5% (n = 223) of patients, with 42.0% (n = 145) undergoing uniportal VATS and 22.5% (n = 78) undergoing multiportal VATS. Thoracotomy was performed in the remaining 35.5% (n = 123).

To account for potential differences between thoracotomy and VATS procedures, a subgroup analysis was conducted. The occurrence of postoperative pneumonia and other complications was compared between patients undergoing thoracotomy (n = 123) and VATS (n = 223). While VATS was associated with a shorter median hospital stay (*p* = 0.023), no statistically significant differences were observed in the rates of pneumonia (*p* = 0.312) or overall postoperative complications (*p* = 0.455) between the two groups. These findings suggest that while surgical approach influences certain recovery metrics, its impact on postoperative pneumonia and broader complications in this cohort was not significant.

### 3.1. Tumour Characteristics

Adenocarcinoma was the most common tumour type, affecting 47.8% (n = 165) of patients, followed by squamous cell carcinoma in 36.4% (n = 126). Tumour staging revealed the highest prevalence in stage T1 (58.4%, n = 202), followed by stage T2 (33.8%, n = 117). Advanced stages T3 and T4 were less frequent, affecting 6.4% (n = 22) and 1.4% (n = 5), respectively. Lymph node staging predominantly showed N0 (74.0%, n = 256), with N1 and N2 each observed in 13.0% (n = 45). Detailed demographic and clinical characteristics are summarised in Table 1.

### 3.2. Premedication

The most frequently used premedication was benzodiazepines, administered to 38.5% (n = 133) of patients, followed by atropine (33.2%, n = 115), antihistamines (18.5%, n = 64), and analgesics (9.8%, n = 34).

Significant trends were observed among the premedication subgroups. Patients receiving antihistamines had a significantly higher median age than other groups (*p* < 0.001), while benzodiazepine recipients included a higher proportion of females (*p* = 0.044). No other statistically significant demographic or clinical differences were identified among the premedication subgroups.

Premedication groups did not significantly differ in terms of operative duration (*p* = 0.572), hospital stay length (*p* = 0.233), pneumonia occurrence (*p* = 0.645), or other postoperative complications (*p* = 0.805). Details are provided in Table 2 and Table 3.

### 3.3. Postoperative Pneumonia and Other Complications

**Figure 1:**
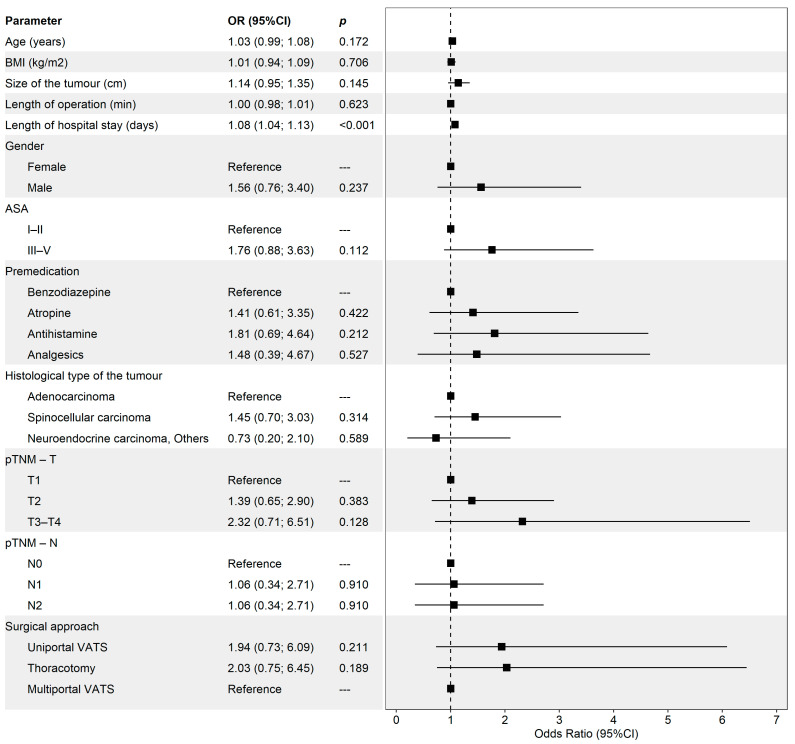
Analysis of the relationship of selected risk factors (intraoperative and postoperative outcomes) and the occurrence of pneumonia. The reference group was selected as the most frequent category and/or the category with the highest estimate of the odds of pneumonia. The figure presents odds ratio (OR) with the 95% confidence interval (95% CI) and the *p*-value of its test of significance.

Pneumonia occurred in 10.7% (n = 37) of patients. Figure 1 presents the detailed analysis of risk factors associated with pneumonia. A longer hospital stay was significantly associated with higher odds of pneumonia (OR [95% CI]: 1.08 [1.04–1.13], *p* < 0.001). No other selected risk factors were significantly associated with pneumonia.

**Figure 2:**
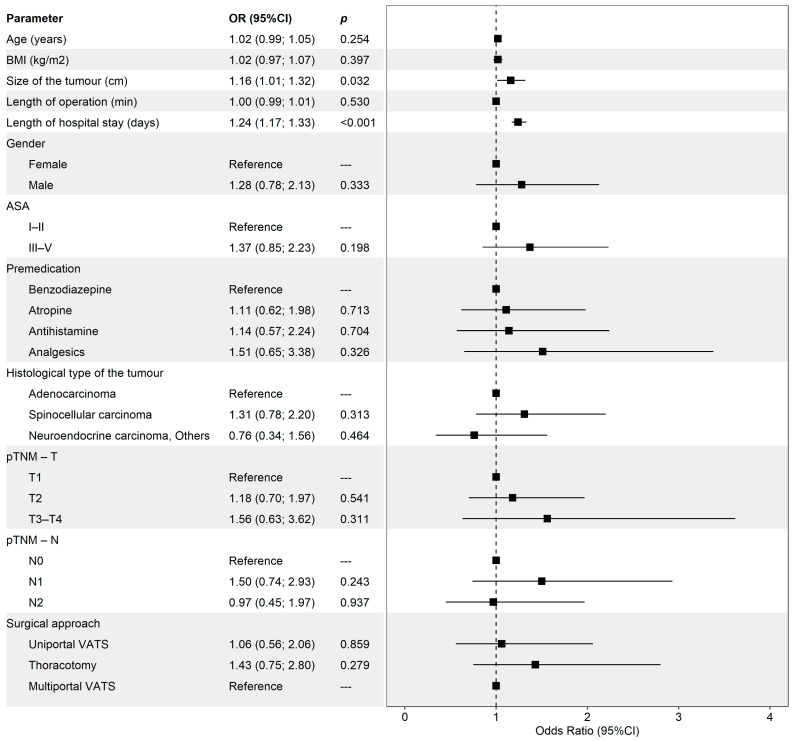
Analysis of the relationship of selected risk factors (intraoperative and postoperative outcomes) and the occurrence of postoperative complications (evaluated as Clavien–Dindo I–V). The reference group was selected as the most frequent category and/or the category with the highest estimate of the odds of postoperative complications. The figure presents the odds ratio (OR) with a 95% confidence interval (95% CI) and the *p*-value of its test of significance.

Postoperative complications (Clavien–Dindo grades I–V) occurred in 26.0% (n = 90) of patients. Tumour size and hospital stay duration were significantly associated with complications. Larger tumours were linked to higher odds of complications (OR [95% CI]: 1.16 [1.01–1.32], *p* = 0.032), and longer hospital stays also increased the odds (OR [95% CI]: 1.24 [1.17–1.33], *p* < 0.001). No other significant associations were identified. Figure 2 illustrates these findings.

## 4. Discussion

Postoperative pneumonia remains a critical concern in thoracic surgery, particularly for patients undergoing lung cancer resection, as it significantly impacts morbidity and mortality. Well-documented risk factors, such as age, BMI, ASA classification, surgical approach, and type of lung resection, are known to contribute to postoperative complications, including pneumonia [19,20,21,22,23]. However, the complex interplay between premedication and its effect on postoperative pneumonia and broader complications necessitates further exploration.

We acknowledge that combining thoracotomy and VATS procedures in the primary analysis could introduce bias due to their differing impacts on pain levels, respiratory recovery, and overall outcomes. Our subgroup analysis indicated no significant differences in postoperative pneumonia or complication rates between the two surgical approaches, which aligns with prior research suggesting that minimally invasive techniques primarily improve short-term recovery metrics, such as hospitalisation duration, without markedly altering complication rates. Nonetheless, we recognise this as a limitation and recommend future studies with larger sample sizes to further delineate the influence of surgical approach on postoperative outcomes.

Although limited research in thoracic surgery directly addresses the role of premedication, studies in other patient populations offer important insights. Choi et al. highlighted that polypharmacy and inappropriate medication use significantly increase the risk of post-discharge institutionalisation and infections in geriatric cancer patients. These findings underscore the need for comprehensive medication reviews, particularly in vulnerable populations, to minimise these risks [13]. Similarly, McDonald et al. demonstrated a strong and linear relationship between the number of preoperative medications and postoperative complications in patients with fragility hip fractures, with the risk plateauing after eight medications. Notably, specific medication types, such as those for cardiac, pulmonary, or diabetic conditions, were identified as particularly high-risk [14]. These observations suggest that preoperative medication management could influence surgical outcomes in various contexts, including thoracic surgery.

Our study, conducted on 346 patients undergoing lobectomy for non-small cell lung cancer (NSCLC), investigated the role of preoperative clinical management, specifically premedication, in influencing postoperative complications. Despite an overall postoperative complication rate of 26.0%, with pneumonia occurring in 10.7%, premedication did not emerge as a statistically significant risk factor for pneumonia or other complications. These findings highlight the need for a more nuanced understanding of how premedication interacts with postoperative outcomes.

### 4.1. Central Findings and Clinical Implications

A key finding of our study was that premedication—comprising atropine, benzodiazepines, and antihistamines—were not significantly associated with the occurrence of postoperative complications. This result contrasts with findings from previous studies, such as those by Paul et al. [24] and Lee et al. [15], which suggested that atropine, particularly in elderly populations, could increase the risk of pneumonia. The lack of significance in our study may reflect differences in patient demographics, perioperative practices, or sample size limitations.

Variability in premedication regimens within our study cohort represents a potential limitation when interpreting these findings. The small size of specific subgroups, coupled with this variability, reduces the statistical power to detect significant associations. While this heterogeneity limits the robustness of our conclusions, it also highlights the need for future studies to standardise premedication protocols and evaluate their impact on perioperative outcomes in larger and more diverse populations.

Our regression models found tumour size and the length of hospital stay to be significantly associated with postoperative complications. Larger tumours were associated with higher odds of complications, a finding that is consistent with the results of previous research conducted by Al-Ameri et al., Paul et al., and Batihan et al. [22,24,25]. This relationship likely reflects the technical challenges and increased tissue disruption involved in the resection of larger tumours. These findings can inform individual practitioners by emphasising the importance of careful preoperative planning and postoperative monitoring, particularly in patients with larger tumours. Moreover, the observed association between prolonged hospital stays and complications suggests a bidirectional relationship, where complications both prolong hospitalisation and result from extended stays [25].

While our study did not identify a significant association between premedication regimens and postoperative complications, this outcome highlights the complexity of perioperative risk factors and underscores the importance of further research. The exploratory nature of our findings provides a valuable foundation for future studies aimed at standardising preoperative care and investigating broader clinical factors influencing postoperative outcomes.

Expanding research efforts to include multi-centre, prospective studies will be critical for evaluating the interplay between premedication regimens and postoperative outcomes in thoracic surgery. By integrating medication management into preoperative assessments, future studies could confirm whether these findings are consistent across surgical specialties or represent unique considerations for specific patient populations.

Additionally, our study offers practical implications for clinical practice. Although premedication did not show a significant association with postoperative pneumonia, the identified risk factors, such as tumour size and hospital stay, can help clinicians better stratify risk and plan interventions. While the regression models developed in this study may not yet be ready for widespread clinical implementation, they can be refined through future prospective studies and adapted into clinical practice for identifying high-risk patients.

### 4.2. Discrepancies with the Literature

Our study revealed no statistically significant correlation between established risk factors—including age, body mass index (BMI), American Society of Anesthesiologists (ASA) classification, and surgical approach—and the incidence of postoperative pneumonia or associated complications. This finding diverges from the extensive body of literature that has historically positioned these variables as critical predictors of postoperative respiratory outcomes [6,7,8,9,10,13,24]. While previous research has consistently characterised advanced age as a prominent risk factor for pneumonia [26,27], our cohort did not substantiate this association. This discrepancy likely stems from methodological limitations inherent in our study design, including its retrospective nature, single-centre configuration, and potential underrepresentation of high-risk patient subgroups. Notably, this variability is not unprecedented, as demonstrated by Ogawa et al. [28], whose findings similarly failed to identify age as a significant predictor in their patient population.

Similarly, while obesity is typically associated with increased surgical risks, our findings align with the “obesity paradox” noted by Tulinský et al. [29,30], suggesting that obesity may have a protective effect in certain thoracic surgery populations. This counterintuitive observation challenges traditional assumptions, emphasising the need for further research into the complex role of obesity in postoperative outcomes.

The absence of a significant relationship between operative duration and complications in our cohort is another point of divergence from the literature, where prolonged surgery is often associated with increased risks of complications [31,32]. This divergence could reflect variations in surgical techniques, expertise, or perioperative care protocols at our institution, which may differ from practices reported in larger, multi-centre studies.

While our study did not identify associations with these well-established risk factors, we believe that the overall findings still offer meaningful insights into the role of premedication in postoperative pneumonia. However, it is important to note that the study was not specifically powered to detect associations with all known risk factors, highlighting the need for further research to validate these preliminary findings in larger, more diverse cohorts.

### 4.3. Strengths, Limitations and Future Directions

Our study has several strengths, including its focus on a specific patient cohort (lobectomy for NSCLC) and its detailed evaluation of premedication and clinical outcomes. However, there are important limitations to consider. The retrospective design of the study prevents us from making causal inferences, and the single-centre nature of the study may limit the generalisability of our findings. Additionally, the relatively small sample size for specific subgroups, such as the mortality cohort, reduces the statistical power of the analysis.

A further limitation of this study is the absence of a formal power calculation. This limitation arises from the retrospective nature of the analysis, which relied on an available dataset encompassing all eligible patients within the study period. Although the total sample size of 346 patients is consistent with similar studies in this field and sufficient for exploratory analyses, the risk of Type II errors, particularly in smaller subgroups, must be acknowledged. In particular, the relatively low incidence of post-operative pneumonia (10%) may have limited the statistical power for detecting associations in this subgroup, highlighting the need for larger, prospective studies to validate these findings. Future studies should incorporate power calculations in their design to ensure adequate sample sizes for detecting clinically meaningful differences.

The selection of variables in our analysis was constrained by the availability of data within the hospital’s information system. Consequently, potentially relevant clinical, socioeconomic, or patient-reported variables may have been excluded, introducing the risk of residual confounding. While we focused on premedication and its relationship to clinical outcomes, the exclusion of variables such as preoperative pulmonary function or comprehensive comorbidity indices may have influenced the findings. Future studies should aim to integrate more comprehensive datasets, potentially leveraging multi-centre or registry-based designs, to capture a broader range of clinically relevant factors and mitigate this limitation.

The inclusion of premedication type in the risk model represents both a strength and a limitation of our analysis. By accounting for the heterogeneity in premedication agents, we aimed to identify potential differential effects on clinical outcomes, including testing the hypothesis that atropine might reduce stagnation pneumonia by limiting postoperative salivation. However, this approach introduces additional complexity, particularly in the context of non-randomised allocation and the retrospective nature of the study. Future prospective research is needed to validate these findings and explore the mechanistic pathways by which specific premedication agents may influence perioperative outcomes.

Given the retrospective nature of the study, we acknowledge that selection bias may have influenced the choice of premedication. While our regression models provide useful insights into risk factors, their application in clinical practice is limited by the exploratory nature of the data and the need for further validation in larger, prospective cohorts. Future research should aim to validate these findings and refine the models in a more diverse patient population, incorporating multi-centre studies to enhance the generalisability of the results. In particular, further investigation into the impact of premedication on vulnerable subgroups, such as elderly patients, is essential to developing more targeted perioperative care strategies.

## 5. Conclusions

In conclusion, our study provides preliminary evidence that premedication may not significantly influence the risk of postoperative pneumonia or overall complications in patients undergoing lobectomy for non-small cell lung cancer. Similarly, other clinical variables, such as age, ASA classification, BMI, and surgical approach, also do not appear to increase these risks. However, our findings highlight the significance of tumour size as a critical determinant of complications and hospitalisation duration in predicting postoperative outcomes.

The limited sample size of our study represents a significant constraint, and the observed effect sizes suggest that detecting statistically significant associations would require a much larger cohort. As such, this study should be regarded as exploratory, serving primarily to generate hypotheses for future research rather than to draw definitive conclusions.

## Figures and Tables

**Table 1:** Characteristics of the study population.

	Total, n = 346
Age, years, median (IQR)	69 (63; 73)
BMI (kg/m^2^), median (IQR)	27.9 (24.8; 31.2)
Size of the tumour, cm, median (IQR)	2.5 (2.0; 4.0)
Length of operation, min, median (IQR)	90 (70; 110)
Length of hospital stay, days, median (IQR)	11 (8; 13)
Gender, n (%)	
Male	212 (61.3)
Female	134 (38.7)
ASA, n (%)	
I–II	174 (50.3)
III	169 (48.8)
IV–V	3 (0.9)
Premedication, n (%)	
Benzodiazepine	133 (38.5)
Atropine	115 (33.2)
Antihistamine	64 (18.5)
Analgesics	34 (9.8)
Histological type of the tumour, n (%)	
Adenocarcinoma	165 (47.8)
Spinocellular carcinoma	126 (36.4)
Neuroendocrine carcinoma	32 (9.2)
Others	23 (6.6)
pTNM—T, n (%)	
T1	202 (58.4)
T2	117 (33.8)
T3	22 (6.4)
T4	5 (1.4)
pTNM—N, n (%)	
N0	256 (74.0)
N1	45 (13.0)
N2	45 (13.0)
Surgical approach, n (%)	
Uniportal VATS	145 (42.0)
Thoracotomy	123 (35.5)
Multiportal VATS	78 (22.5)
Pneumonia, n (%)	
Yes	37 (10.7)
No	309 (89.3)
Clavien–Dindo classification, n (%)	
Grade 0	256 (74.0)
Grade I–II	55 (15.9)
Grade III–IV	23 (6.6)
Grade V	12 (3.5)

IQR—interquartile range; BMI—body mass index; ASA—American Society of Anesthesiologists classification; pTNM—pathological TNM staging (T—tumour; N—nodus; M—metastasis).

**Table 2:** Demographic and clinical characteristics of subgroups according to premedication.

	Benzodiazepine, n = 133	Atropine, n = 115	Antihistamine, n = 64	Analgesics, n = 34	*p* ^a^
Age, years, median (IQR)	67 (61; 71)	68 (63; 73)	73 (68; 76)	68 (64; 73)	<0.001 ^b^
BMI (kg/m^2^), median (IQR)	27.8 (24.4; 31.1)	27.5 (24.4; 31.0)	28.4 (26.4; 31.8)	29.1 (26.8; 31.1)	0.194
Size of the tumour, cm, median (IQR)	3.0 (1.6; 4.0)	2.5 (2.0; 3.8)	2.5 (2.0; 3.6)	2.8 (2.0; 3.4)	0.961
Gender, n (%)					0.044
Male	70 (52.6)	79 (68.7)	39 (60.9)	24 (70.6)	
Female	63 (47.4)	36 (31.3)	25 (39.1)	10 (29.4)	
ASA, n (%)					0.100
I–II	69 (51.9)	65 (56.5)	24 (37.4)	16 (47.1)	
III	63 (47.4)	50 (43.5)	39 (60.9)	17 (50.0)	
IV–V	1 (0.7)	---	1 (1.7)	1 (2.9)	
Histological type of the tumour, n (%)					0.197
Adenocarcinoma	73 (54.9)	46 (40.0)	31 (48.4)	15 (44.2)	
Spinocellular carcinoma	39 (29.3)	53 (46.1)	22 (34.5)	12 (35.3)	
Neuroendocrine carcinoma	12 (9.0)	11 (9.6)	4 (6.2)	5 (14.7)	
Others	9 (6.8)	5 (4.3)	7 (10.9)	2 (5.8)	
pTNM—T, n (%)					0.674
T1	73 (54.9)	66 (57.4)	39 (60.9)	24 (70.6)	
T2	47 (35.3)	42 (36.5)	20 (31.2)	8 (23.5)	
T3	9 (6.8)	7 (6.1)	4 (6.2)	2 (5.9)	
T4	4 (3.0)	---	1 (1.7)	---	
pTNM—N, n (%)					0.984
N0	101 (75.9)	83 (72.2)	48 (75.0)	24 (70.6)	
N1	17 (12.8)	16 (13.9)	7 (10.9)	5 (14.7)	
N2	15 (11.3)	16 (13.9)	9 (14.1)	5 (14.7)	
Surgical approach, n (%)					0.414
Uniportal VATS	52 (39.1)	55 (47.8)	23 (36.0)	15 (44.2)	
Thoracotomy	49 (36.8)	40 (34.8)	21 (32.8)	13 (38.2)	
Multiportal VATS	32 (24.1)	20 (17.4)	20 (31.2)	6 (17.6)	

^a^ The *p*-value of Kruskal–Wallis test or Chi-square test of independence for contingency tables; ^b^ post hoc analysis (Dunn’s method)—homogeneous groups: (antihistamine) and (atropine, analgesic, benzodiazepine). The relative frequencies are calculated as column relative frequencies. IQR—interquartile range; BMI—body mass index; ASA—American Society of Anaesthesiologists classification; pTNM—pathological TNM staging (T—tumour; N—nodus; M—metastasis)

**Table 3:** The intraoperative and post-operative outcomes of subgroups according to premedication.

	Benzodiazepine, n = 133	Atropine, n = 115	Antihistamine, n = 64	Analgesics, n = 34	*p* ^a^
Length of operation, min, median (IQR)	90 (70; 105)	90 (75; 110)	90 (74; 110)	90 (70; 110)	0.572
Length of hospital stay, days, median (IQR)	10 (8; 13)	11 (8; 13)	11 (9; 13)	11 (8; 13)	0.233
Pneumonia, n (%)	11 (8.3)	13 (11.3)	9 (14.1)	4 (11.8)	0.645
Postoperative complications, n (%)	32 (24.1)	30 (26.1)	17 (26.6)	11 (32.4)	0.805
Clavien–Dindo classification, n (%)					0.893
Grade 0	101 (75.9)	85 (73.9)	47 (73.4)	23 (67.6)	
Grade I–II	19 (14.3)	21 (18.3)	10 (15.6)	5 (14.7)	
Grade III–IV	8 (6.0)	7 (6.1)	4 (6.3)	4 (11.8)	
Grade V	5 (3.8)	2 (1.7)	3 (4.7)	2 (5.9)	0.430

^a^ The *p*-value of Kruskal–Wallis test or Chi-square test of independence for contingency tables. The relative frequencies are calculated as column relative frequencies. IQR—interquartile range.

## Data Availability

The data are available upon request from the corresponding author.

## References

[1] McIntyre A., Ganti A.K. (2017). Lung cancer-A global perspective. J. Surg. Oncol..

[2] Cancer Today. https://gco.iarc.fr/.

[3] Barta J.A., Powell C.A., Wisnivesky J.P. (2019). Global Epidemiology of Lung Cancer. Ann. Glob. Health.

[4] Bade B.C., Dela Cruz C.S. (2020). Lung Cancer 2020. Clin. Chest. Med..

[5] Hoy H., Lynch T., Beck M. (2019). Surgical Treatment of Lung Cancer. Crit. Care Nurs. Clin. N. Am..

[6] Manerikar A., Querrey M., Cerier E., Kim S., Odell D.D., Pesce L.L., Bharat A. (2021). Comparative Effectiveness of Surgical Approaches for Lung Cancer. J. Surg. Res..

[7] Bendixen M., Jørgensen O.D., Kronborg C., Andersen C., Licht P.B. (2016). Postoperative pain and quality of life after lobectomy via video-assisted thoracoscopic surgery or anterolateral thoracotomy for early stage lung cancer: A randomised controlled trial. Lancet Oncol..

[8] Mercier O. (2022). VATS versus open thoracotomy for lung cancer resection: Is the game still running?. Eur. J. Cardiothorac. Surg..

[9] Lim E., A Harris R., E McKeon H., Batchelor T.J., Dunning J., Shackcloth M., Anikin V., Naidu B., Belcher E., Loubani M. (2022). Impact of video-assisted thoracoscopic lobectomy versus open lobectomy for lung cancer on recovery assessed using self-reported physical function: VIOLET RCT. Health Technol. Assess..

[10] Gao H.-J., Jiang Z.-H., Gong L., Ma K., Ren P., Yu Z.-T., Wei Y.-C. (2019). Video-Assisted Vs Thoracotomy Sleeve Lobectomy for Lung Cancer: A Propensity Matched Analysis. Ann. Thorac. Surg..

[11] Couñago F., Luna J., Guerrero L.L., Vaquero B., Guillén-Sacoto M.C., González-Merino T., Taboada B., Díaz V., Rubio-Viqueira B., Díaz-Gavela A.A. (2019). Management of oligometastatic non-small cell lung cancer patients: Current controversies and future directions. World J. Clin. Oncol..

[12] Freeman B.S., Berger J.S. (2014). Anesthesiology Core Review. Part 1, Basic Exam.

[13] McDonald C.L., Cohen B.H., Medina Pérez G., Modest J.M., Kuris E.O., Born C. (2022). Pre-Operative Medications as a Predictor for Post-Operative Complications Following Geriatric Hip Fracture Surgery. Geriatr. Orthop. Surg. Rehabil..

[14] Choi K.S., Jeong Y.M., Lee E., Kim K.I., Yee J., Lee B.K., Chung J.E., Rhie S.J., Gwak H.S. (2018). Association of pre-operative medication use with post-surgery mortality and morbidity in oncology patients receiving comprehensive geriatric assessment. Aging Clin. Exp. Res..

[15] Lee C.-Y., Cheng Y.-D., Cheng W.-Y., Tsai T.-H., Huang K.-H. (2020). The Prevalence of Anticholinergic Drugs and Correlation with Pneumonia in Elderly Patients: A Population-Based Study in Taiwan. Int. J. Environ. Res. Public Health.

[16] Zhang H.-X., Shen Y., Chen J., Zhang L., Lin W. (2020). Risk Factors of Pulmonary Complications After Minimally Invasive Surgery for Elderly Patients with Vertebral Compression Fractures. Ther. Clin. Risk Manag..

[17] Seely A.J., Ivanovic J., Threader J., Al-Hussaini A., Al-Shehab D., Ramsay T., Gilbert S., Maziak D.E., Shamji F.M., Sundaresan R.S. (2010). Systematic classification of morbidity and mortality after thoracic surgery. Ann. Thorac. Surg..

[18] Jammer I., Wickboldt N., Sander M., Smith A., Schultz M.J., Pelosi P., Leva B., Rhodes A., Hoeft A., Walder B. (2015). Standards for definitions and use of outcome measures for clinical effectiveness research in perioperative medicine. Eur. J. Anaesthesiol..

[19] Schussler O., Alifano M., Dermine H., Strano S., Casetta A., Sepulveda S., Chafik A., Coignard S., Rabbat A., Regnard J.-F. (2006). Postoperative Pneumonia after Major Lung Resection. Am. J. Respir. Crit. Care Med..

[20] Detillon D.D.E.M.A., Veen E.J. (2018). Postoperative Outcome After Pulmonary Surgery for Non-Small Cell Lung Cancer in Elderly Patients. Ann. Thorac. Surg..

[21] Yao L., Luo J., Liu L., Wu Q., Zhou R., Li L., Zhang C. (2021). Risk factors for postoperative pneumonia and prognosis in lung cancer patients after surgery. Medicine.

[22] Al-Ameri M., Bergman P., Franco-Cereceda A., Sartipy U. (2018). Video-assisted thoracoscopic versus open thoracotomy lobectomy: A Swedish nationwide cohort study. J. Thorac. Dis..

[23] Guerrera F., Lyberis P., Lausi P.O., Cristofori R.C., Giobbe R., Molinatti M., Filosso P.L., Curcio C., Crisci R., Ruffini E. (2021). Does morbid obesity influence perioperative outcomes after video-assisted thoracic surgery (VATS) lobectomy for non-small cell lung cancer? Analysis of the Italian VATS group registry. Surg. Endosc..

[24] Paul K.J., Walker R.L., Dublin S. (2015). Anticholinergic Medications and Risk of Community-Acquired Pneumonia in Elderly Adults: A Population-Based Case-Control Study. J. Am. Geriatr. Soc..

[25] Batihan G., Ceylan K.C., Usluer O., Kaya Ş.Ö. (2020). Video-Assisted Thoracoscopic Surgery vs Thoracotomy for Non-Small Cell Lung Cancer Greater Than 5 cm: Is VATS a feasible approach for large tumors?. J. Cardiothorac. Surg..

[26] Simonsen D.F., Søgaard M., Bozi I., Horsburgh C.R., Thomsen R.W. (2015). Risk factors for postoperative pneumonia after lung cancer surgery and impact of pneumonia on survival. Respir. Med..

[27] Lee J.Y., Jin S.-M., Lee C.-H., Lee B.J., Kang C.-H., Yim J.-J., Yang S.-C., Yoo C.-G., Han S.K., Kim J.H. (2011). Risk factors of postoperative pneumonia after lung cancer surgery. J. Korean Med. Sci..

[28] Ogawa F., Wang G., Matsui Y., Hara H., Iyoda A., Satoh Y. (2013). Risk factors for postoperative complications in the elderly with lung cancer. Asian Cardiovasc. Thorac. Ann..

[29] Tulinský L., Sengul I., Ihnát P., Ostruszka P., Toman D., Guňková P., Pelikán A., Sengul D. (2022). Obesity in cases undergoing the surgical procedure of lung lobectomy: Risk or benefit?. Rev. Assoc. Med. Bras..

[30] Tulinský L., Mitták M., Tomášková H., Ostruszka P., Penka I., Ihnát P. (2018). Obesity paradox in patients undergoing lung lobectomy—Myth or reality?. BMC Surg..

[31] de Angelis P., Tan K.S., Chudgar N.P., Dycoco J., Adusumilli P.S., Bains M.S., Bott M.J., Downey R.J., Huang J., Isbell J.M. (2022). Operative Time Is Associated with Postoperative Complications After Pulmonary Lobectomy. Ann. Surg..

[32] Gómez-Hernández M.T., Forcada C., Varela G., Jiménez M.F. (2022). Operating time: An independent and modifiable risk factor for short-term complications after video-thoracoscopic pulmonary lobectomy. Eur. J. Cardiothorac. Surg..

